# Pathophysiological Effects on Coronary Arteries Following Radiofrequency Ablation: A Comprehensive Review

**DOI:** 10.1111/anec.70021

**Published:** 2025-01-22

**Authors:** Syed Ali Raza Abidi, Afreen Quadri, Muhammad Umer Riaz Gondal, Fatima Hayat, Shafia Naeem, Fawad Talat, Amin Mehmoodi, Jahanzeb Malik

**Affiliations:** ^1^ Department of Medicine Allama Iqbal Teaching Hospital DG Khan Pakistan; ^2^ Department of Medicine Dr. VRK Womens Medical College Aziznagar India; ^3^ Department of Medicine Reading Hospital Philadelphia USA; ^4^ Department of Medicine Army Medical College Rawalpindi Pakistan; ^5^ Department of Medicine University of Health Sciences Lahore Pakistan; ^6^ Department of Medicine King Edward Medical University Lahore Pakistan; ^7^ Department of Medicine Ibn e Seena Hospital Kabul Afghanistan; ^8^ Cardiovascular Analytics Group Islamabad Pakistan

**Keywords:** arrhythmia treatments, coronary artery injury, lesion formation, pathophysiology, radiofrequency ablation

## Abstract

Radiofrequency ablation (RFA) is a safe and effective treatment for patients experiencing ventricular and atrial tachyarrhythmias. While complications after RFA are generally rare, the occurrence of coronary artery (CA) injury, albeit infrequent, can have significant clinical implications. Given the proximity of CAs to common ablation sites, understanding the interplay between RFA and CA perfusion pathophysiology is paramount. Although previous studies have discussed the presentation and outcomes of CA injury post‐ablation, a comprehensive review consolidating the mechanisms of CA injury following RFA remains absent in the cardiology literature. In this review, we conducted an extensive literature search spanning the past three decades to explore the link between the biophysics of RFA and CA perfusion pathophysiology, focusing on injury mechanisms. We delve into RFA lesion pathology, elucidate the mechanisms of CA injury resulting from RFA, and examine factors influencing lesion formation, such as convective cooling and the “shadow effect.” Furthermore, we outline methods to mitigate CA injury post‐RFA and propose novel research avenues to optimize lesion formation and ensure the safety of arrhythmia treatments, particularly in cases where tissue ablation is performed close to CAs.

## Graphical abstract

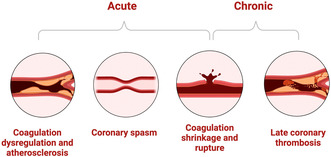

## 1 Introduction

Over more than 30 years, the utilization of catheter radiofrequency ablation (RFA) has evolved from treating simpler arrhythmias like Wolff–Parkinson–White Syndrome and atrioventricular nodal reentrant tachycardia to more complex cases such as atrial fibrillation (AF) and ventricular tachycardia (VT). Generally, RFA demonstrates high efficacy and an outstanding safety profile, with complication rates typically ranging from 1% to 5% according to recent surveys (Cappato et al. 2010; Terasawa et al. 2009). However, these rates can vary depending on the specific arrhythmia being treated and the volume of myocardium ablated. Procedural complications associated with RFA include pulmonary vein stenosis, pericardial effusion, cardiac tamponade, peri‐procedural stroke or transient ischemic attack, atrio‐esophageal fistula, phrenic nerve paralysis, and peripheral vascular issues such as deep vein thrombosis, pseudoaneurysm, and hematoma at the catheter insertion site necessitating transfusion or invasive intervention.

Coronary artery (CA) injury is a rare but serious complication of RFA. Although reported incidence rates are low (0.09%), the proximity of common ablation sites to CAs suggests that CA injury may be underreported, potentially due to undetected pathophysiological changes (Roberts‐Thomson et al. 2009; Schneider et al. 2009). To comprehensively address CA injury after RFA, we delve into general RFA lesion pathology, mechanisms of CA injury post‐RFA, and various factors influencing lesion formation such as convective cooling and the opposing “shadow effect” during arrhythmia treatment. We explore specific procedures and patient sub‐groups associated with heightened CA injury risk. Lastly, we identify areas for further research and development of novel ablation techniques aimed at enhancing safety and procedural efficacy.

## 2 Pathological Changes in Lesions Following RFA

**FIGURE 1:**
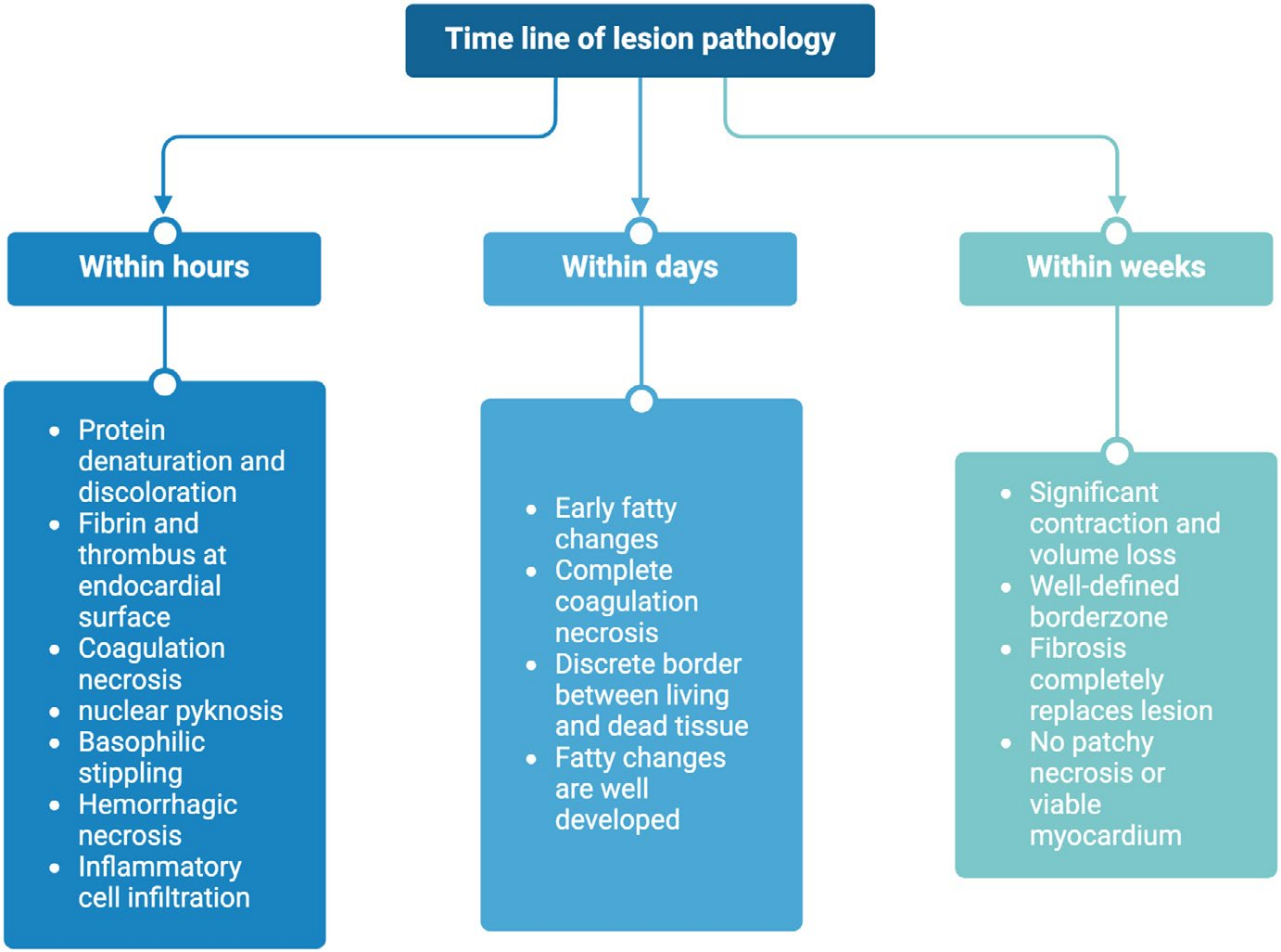
Timeline of the general lesion pathology after RFA.

Understanding the progression of tissue pathology at the radiofrequency (RF) lesion site is crucial for planning safe and effective RFA procedures. Changes at the lesion site become apparent immediately following RFA and continue to evolve over approximately 8 weeks. Upon instantaneous RF delivery, tissue discoloration occurs due to protein denaturation, particularly of myoglobin, resulting in the loss of its red pigment (Haines 2004). Histological examination within hours of RFA reveals a central zone characterized by coagulation necrosis, nuclear pyknosis, and basophilic stippling indicating intracellular calcium overload (Haines 2004). Surrounding this central necrotic area is a hemorrhagic transition zone (granulation tissue), accompanied by infiltrating mononuclear inflammatory cells, edema, and increased tissue thickness (Wittkampf, Hauer, and Robles de Medina 1989; Huang, Graham, and Wharton 1988; Ren et al. 2001). Within 4–5 days, complete coagulation necrosis and early fatty changes become evident in the central lesion zone, while the transition zone disappears. By the end of the first week, fatty changes progress, and by Week 8, fibrosis has entirely replaced the lesion (Huang et al. 1987). Figure 1 illustrates the timeline of lesion pathophysiology after RFA.

**FIGURE 2:**
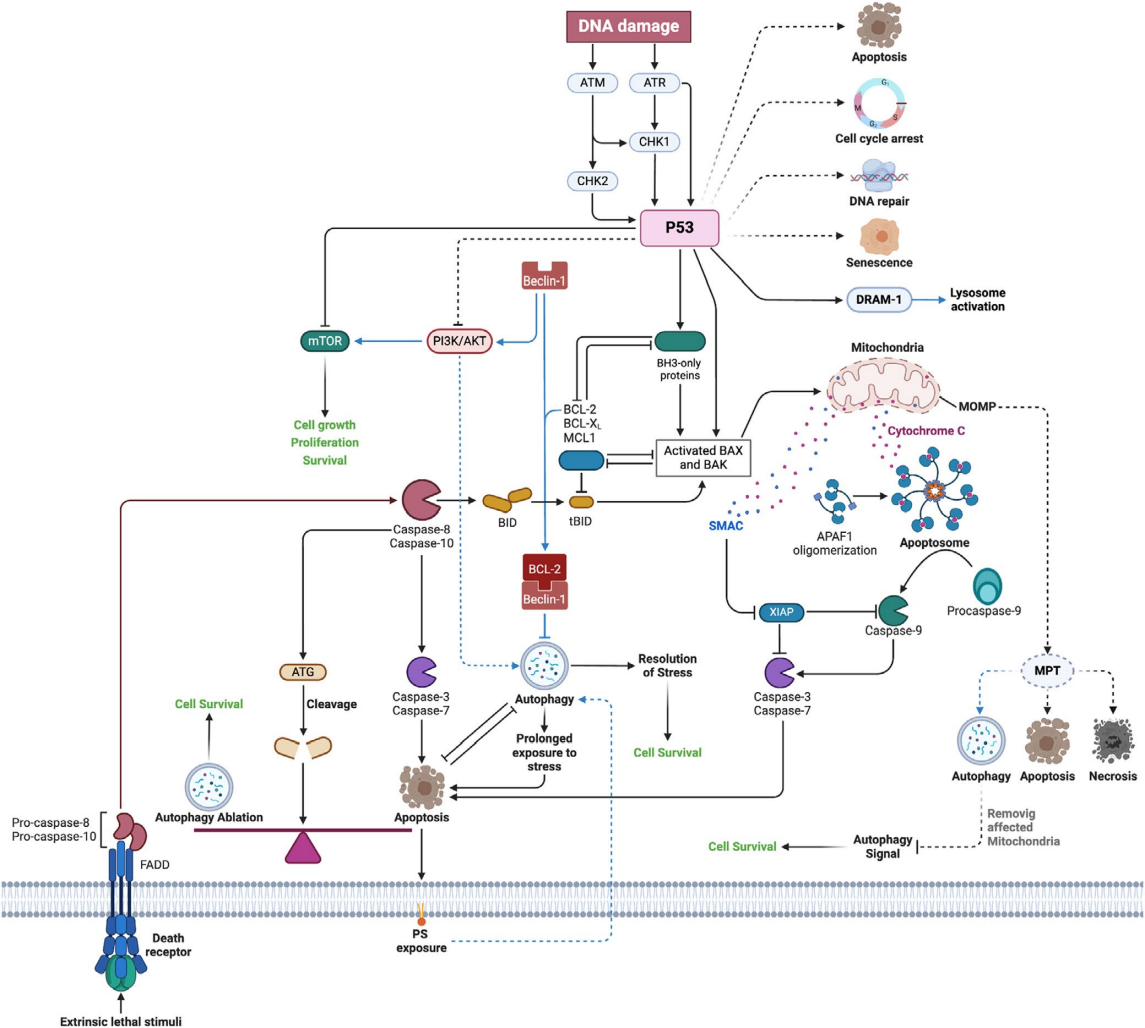
Crosstalk between apoptosis and autophagy in RFA.

After RFA, cellular responses such as apoptosis and autophagy play significant roles in the subsequent tissue changes. Apoptosis, also known as programmed cell death, is a highly regulated process involving the activation of specific pathways leading to cell fragmentation and removal. Following RFA, apoptosis can occur in the ablated tissue as a response to the thermal and mechanical stress induced by the procedure. Autophagy, on the other hand, is a cellular process involved in the degradation and recycling of cellular components to maintain cellular homeostasis. After RFA, autophagy may be upregulated as a protective mechanism to remove damaged organelles and proteins caused by the thermal injury. Both apoptosis and autophagy contribute to the overall tissue remodeling and healing process following RFA, and understanding these cellular mechanisms is crucial for optimizing treatment outcomes and minimizing adverse effects (Figure 2).

In general, the degree of acute injury at the RF lesion is thought to play a critical role in determining the success or failure of ablation at an arrhythmogenic focus. However, various other factors can also impact efficacy. First, thermal latency results in a prolonged increase in tissue temperature that may persist for over 30 s after RF delivery cessation. This prolonged temperature elevation explains how an arrhythmia may permanently disappear several hours after what was initially perceived as an unsuccessful RFA (Wittkampf et al. 1996; Takahashi et al. 2002). Second, the growth of the ablation lesion has been proposed as a factor contributing to long‐term freedom from atrial fibrillation (AF) following an early recurrence of the arrhythmia (Oral et al. 2002; Nath et al. 1994). Lastly, the arrhythmogenic substrate may regain function within a few weeks following what was initially deemed a successful RFA, as inflammatory and necrotic tissue regress (Langberg et al. 1992, 1993). Therefore, various phenomena influencing the RFA lesion have implications for RFA outcomes.

## 3 Mechanisms Underlying CA Damage Induced by RFA

Considering the proximity of CAs to commonly ablated sites, it is reasonable to suggest that RFA may not only affect surrounding tissues but also compromise vascular integrity and function (Roberts‐Thomson et al. 2009). While the presentation and outcomes of CA injury following RFA in adults have been well documented, specific mechanisms of such injury can vary from case to case, as discussed by Roberts‐Thomson et al. However, a comprehensive summary of these mechanisms has yet to be consolidated in the cardiology literature. Drawing from our review of case reports and case series, we outline how RFA acutely and subacutely causes CA injury.

**FIGURE 3:**
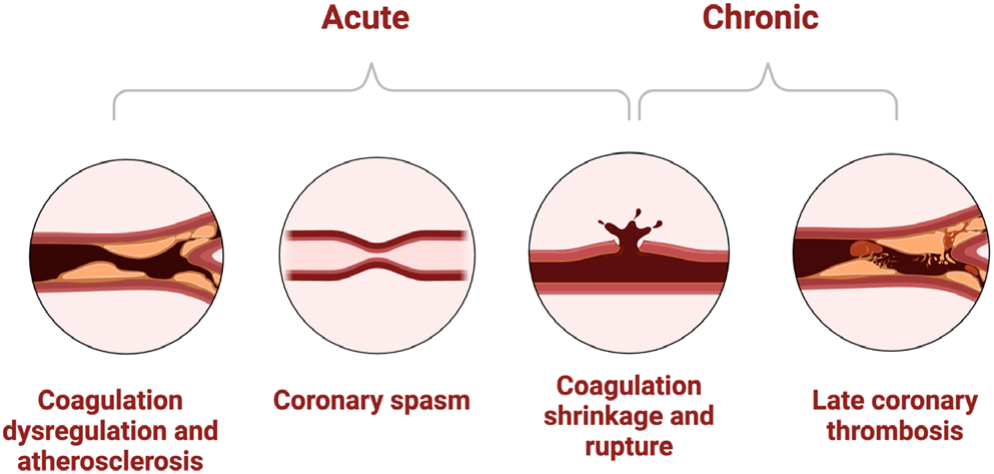
Mechanisms of coronary artery injury post‐RFA.

In the acute phase, RF energy can lead to coronary spasm, direct trauma to the vessels, and thromboembolism. Among these, spasm is believed to be the most common mechanism of coronary injury following RFA, particularly when RF energy is applied in the coronary sinus or on the epicardium (Pons et al. 1997). Evidence supporting coronary spasm after RFA includes reports of presumed acute arterial occlusions responsive to nitrates, despite angiographically normal vessels (Solomon et al. 1993; Simon and Gill 2003; Bardy et al. 1988; Lesh et al. 1992). The mechanism behind this spasm is attributed to RF‐induced increases in autonomic activity at nerve terminals within the densely innervated left atrium (Yamashita et al. 2007; Pauza et al. 2002; Pauziene and Pauza 2003; Saburkina et al. 2010) (Figure 3).

RF energy also induces functional and morphological damage to the CA media and endothelium. This direct damage compromises the vessel's ability to regulate vascular tone and coagulation, thereby increasing the likelihood of nidus formation leading to acute or subacute thrombosis. In a porcine model, epicardial RF lesions created near major CAs were associated with both disruption of the vascular wall and significant alterations in vascular tone (Demaria et al. 2003). Application of RF within 1 mm of the CA resulted in a notable decrease in endothelium‐independent contraction to potassium chloride and endothelium‐dependent relaxation to bradykinin. Histological examination revealed disruption of the vascular wall up to 5 mm from the RF application site, corroborating these functional findings. Consequently, Sosa et al. have advised against applying RF to an epicardial site within 12 mm of a major CA (Sosa, Scanavacca, and d'Avila 2001).

Another mechanism proposed for RFA‐induced CA damage involves heat‐induced collagen shrinkage and subsequent vessel narrowing (Aoyama et al. 2005). It is understood that the degree of CA stenosis correlates with the extent of heat‐induced denaturation of collagen fibers in the vessel wall (Gorisch and Boergen 1982; Kang, Resar, and Humphrey 1995). Studies, including one involving nine mongrel dogs, have demonstrated that replacement of the coronary arterial media with proliferating extracellular matrix leads to severe hyperplasia and intravascular thrombosis (D'Avila et al. 2002). In these experiments, researchers also compared the effects of RFA with the orientation of the lesion line and its proximity to CAs. The extent of vessel wall injury depended on how the ablation line intersected the CA: RFA delivered adjacent and parallel to the artery resulted in lesions primarily limited to the media, whereas RFA delivered directly and perpendicularly to the artery led to severe intimal hyperplasia and intravascular thrombosis. Thus, the heat from the catheter tip and its orientation relative to the CA are crucial considerations for minimizing intimal hyperplasia and intravascular thrombosis during ablation procedures.

## 4 Balancing the “Heat Sink” and “Shadow Effect”: Evaluating Convective Cooling's Role in Safeguarding CAs and Its Impact on RF Lesion Formation

Lesion size, properties of RF energy, proximity, and orientation to CAs are recognized factors determining the impact of RFA on CAs. Additionally, convective cooling emerges as a significant factor influencing lesion formation. Initially observed in the context of hyperthermia treatments for malignancies, convective cooling arises from the flow of intracardiac and microvascular blood, creating a “heat sink” (Haines 2004). Generally, the susceptibility of CAs to thermal damage decreases as the electrode‐to‐artery distance increases (Haines 2004; D'Avila et al. 2002). Temperature gradients are generated as heat transfers from the electrode tip through the tissue, and the likelihood of thermal injury to CAs diminishes with increasing distance from the catheter tip (Haines and Watson 1989). This thermal model remains valid irrespective of the convective cooling phenomenon (Haines and Watson 1989). In the “heat sink” effect, when an RF electrode is positioned close to a vessel, coronary blood flow within and surrounding the vessel acts as a protective mechanism by impeding significant heating of the vascular endothelium (Chatelain et al. 1995). Consequently, convective cooling may contribute to the scarcity of reported CA complications or observable changes in coronary arteriograms conducted pre‐ and post‐RFA (Hindricks 1993).

However, the protective effect provided by convective cooling during RFA may be constrained by the brief duration of energy delivery and reductions in microvascular perfusion of ablated tissue (Haines and Watson 1989). Ablation procedures conducted in young or small hearts, where narrower vessels result in a less pronounced cooling phenomenon, may heighten susceptibility to CA injury (Bokenkamp et al. 2000). Moreover, pre‐existing narrowed atherosclerotic CAs in patients with coronary artery disease (CAD) face an increased risk of thermal injury. For instance, in a patient undergoing RFA for atrial flutter, an undocumented upstream right CA stenosis, which restricted flow and diminished convective cooling, was believed to be the mechanism underlying CA injury (Ouali et al. 2002).

**FIGURE 4:**
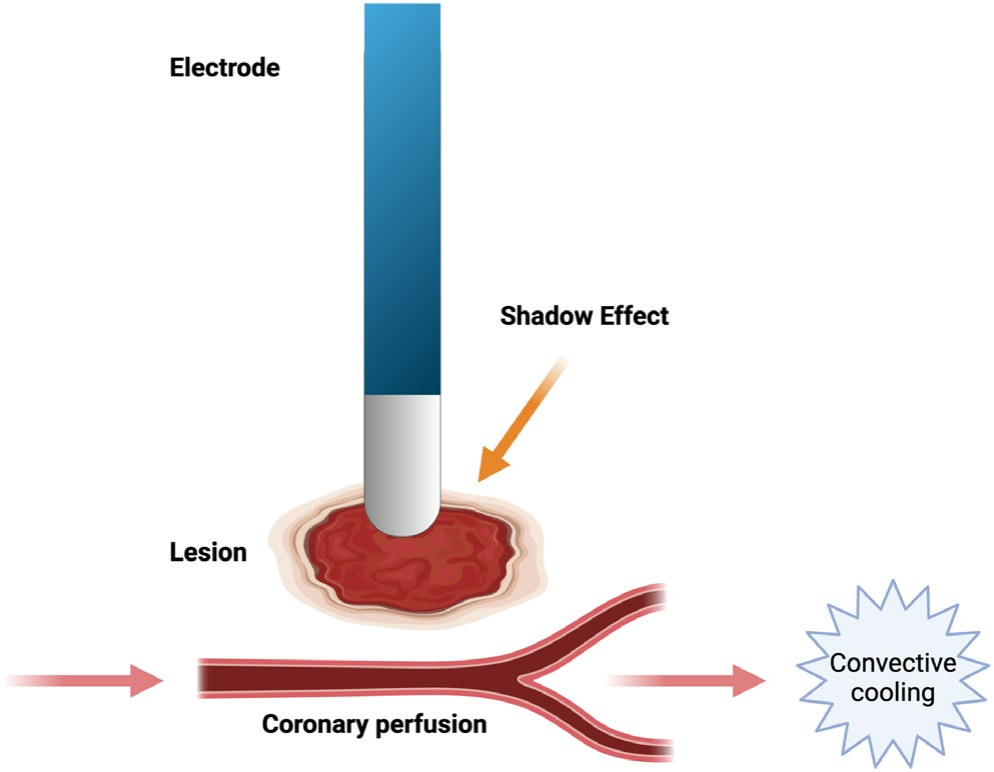
Convective cooling and heat sink effects. Convective cooling provides a protective mechanism for coronary arteries against thermal energy generated during RFA. However, this phenomenon must be carefully balanced with the “shadow effect.” The “shadow effect” can hinder the formation of transmural lesions, maintain conduction through an RF lesion, and consequently prevent a complete conduction block. Thus, both convective cooling and the “shadow effect” should be considered when planning and performing RFA procedures to achieve optimal treatment outcomes.

The cooling effect exerted by small coronary vasculature, although offering less protection against thermal injury compared to larger vessels, can sometimes effectively impede RF lesion formation and reduce ablation efficacy. Fuller and Wood (2003) demonstrated in a rabbit model that even flow through small intramyocardial vessels could prevent the formation of transmural lesions, maintaining conduction through an RF lesion and consequently preventing a complete conduction block. This phenomenon, termed the “shadow effect,” must be carefully considered alongside the benefits of the “heat sink” to enhance the likelihood of an RF lesion effectively eliminating aberrant electrical conduction. These mechanisms are shown in Figure 4.

Comprehending the parameters influencing lesion formation and their correlation with coronary blood flow in both large and small coronary vessels is crucial for ensuring safe and effective ablation procedures. For instance, Fuller et al. highlighted that the extent of protected myocardium surrounding the perfused vessel is linked to arterial flow rate, intramural vascular diameter, and lesion temperature, while electrical conduction through a lesion is influenced by arterial flow rate and the volume of preserved myocardium. Integrating these parameters into pre‐ablation assessments and computational models of lesion formation has the potential to enhance the safety and efficacy of ablation techniques.

## 5 Enhancing Safety and Efficacy of RFA Through Predictive Lesion Dimension Modeling

We have explored several factors influencing the formation of RF lesions and the overall success of RFA. Extensive modeling in experimental tissue systems indicates that some of these factors can be quantified before RF administration to predict lesion size. For instance, in a tissue model of myocardial electrical conductivity, convective cooling was estimated by maximum tip temperature increase and the slope of temperature decay, showing strong correlations with flow rate and predicting lesion dimensions (Huang, Chen, and Roemer 1996; Haemmerich and Saul 2006). Consequently, treatment planning for cardiac RFA could potentially estimate RF lesion dimensions based on target temperature, ablation time, and blood flow (Lai et al. 2004). Incorporating additional measures such as thermal latency, lesion growth, healing processes, the shadow effect, and tip temperature into computational models may further refine predictions of lesion formation, potentially reducing the incidence of CA injury and enhancing the efficacy of RFA (Lai et al. 2004).

## 6 Avoiding CA Damage During RFA

Enhanced protocol strategies, combined with computational assessments, can effectively mitigate CA injury risks during RFA procedures. For instance, minimizing RF energy delivery during ablation is prudent, particularly in smaller patients or those with severe CA stenosis, where critical structures may be near the ablation site (Gorisch and Boergen 1982; Paul et al. 2003; Bertram et al. 2001). Furthermore, understanding a patient's specific coronary anatomy is paramount. During epicardial ablations, the operator must exercise caution when applying RF energy near major CAs vulnerable to RFA‐induced damage. Notably, major CAs often traverse regions close to the right ventricular outflow tract (RVOT). For instance, the left main CA may run within the periphery of potential ablation sites in septal regions of the RVOT near the pulmonary valve. Hence, electrophysiologists should meticulously delineate the anatomical trajectories of major CAs before VT ablation originating from the RVOT (Vaseghi et al. 2006).

Another proposed strategy to mitigate CA injury during RFA is chilled saline irrigation. Thyer et al. showcased in an in vitro ovine heart model that intracoronary irrigation with chilled saline safeguarded the CA endothelium from heat‐induced damage and decreased the likelihood of the ablation lesion spreading to the CA during epicardial RFA (Thyer et al. 2006). Further investigation through in vivo animal studies and subsequent trials is necessary to evaluate the safety and efficacy of this approach.

Fluoroscopic or electroanatomic verification of catheter position and the utilization of smaller tip catheters are simple measures that can be implemented before RFA to mitigate CA complications (Mykytsey et al. 2010). In individuals with known or suspected CAD, angiography could assess the extent of CAD and establish the anatomical proximity between the catheter and coronary vessels. It has been recommended that RF pulses should be administered only if large‐diameter vessels are at least 4 mm away from the ablation catheter (Roberts‐Thomson et al. 2009; D'Avila et al. 2002; Ouali et al. 2002; Mykytsey et al. 2010).

Irrigated ablation is a common technique used to create more effective lesions while minimizing the risk of overheating the tissue during cardiac ablation procedures. The catheter tip in irrigated ablation is cooled through continuous saline infusion, which allows higher energy delivery without causing excessive heating at the surface of the myocardium (Haines and Watson 1989; Chatelain et al. 1995). This cooling effect enables the creation of deeper lesions that can reach arrhythmogenic tissue located deeper in the heart wall. By reducing surface temperatures, the risk of thrombus formation and char buildup is also lowered, making the procedure safer.

However, the depth of the lesion is an important consideration, especially in relation to surrounding structures such as the CAs. Because irrigated ablation creates deeper lesions, there is a higher risk of collateral damage to adjacent tissues (Hindricks 1993; Ouali et al. 2002). This is particularly relevant when ablating near the CAs, as thermal injury to these vessels can lead to serious complications such as CA spasm, thrombosis, or long‐term vascular damage. Understanding the proximity of the CAs to the ablation target is crucial to avoid unintentional damage.

In terms of cooling and tissue response, irrigated ablation maintains tissue temperatures at safer levels while still allowing the ablation to reach the necessary depths for effective treatment of arrhythmias. However, this added cooling requires precise control, as overcooling can lead to excessive tissue injury beyond the intended area (Ouali et al. 2002). Therefore, careful monitoring and mapping are essential during the procedure, particularly when ablating near critical structures like the CAs.

## 7 Future Directions and Conclusion

Cryoablation energies may offer a safer alternative for patients who exhibit ST‐segment elevation during RFA (Spar et al. 2010). In a canine model, cryoablation was compared to RFA near the circumflex CA within the coronary sinus. Histological analysis revealed medial necrosis within the CA with both modalities, but only RFA led to disruption of the elastic lamina and loss of intimal endothelial cells (Aoyama et al. 2005). Intravascular echocardiography and angiography demonstrated CA narrowing with RFA but not with cryoablation. This suggests that cryoablation may be safer than RFA in certain situations when a CA is in proximity (Aoyama et al. 2005).

High‐intensity focused ultrasound (HIFU) represents a newer, extracorporeal lesion‐forming technology capable of creating thermal ablation within a defined focal volume without affecting neighboring tissues such as large blood vessels (Cesario, Mahajan, and Shivkumar 2007). One advantage of HIFU is its precise focus in a targeted tissue region via a remote transducer, inducing molecular vibration and friction that lead to rapid absorptive heating, thermal coagulation, and ultimately necrosis of the targeted region (Hill et al. 1994; Hill and ter Haar 1995; ter Haar 1995; Illing et al. 2005). Additionally, other technologies and the impact of alternate energies such as laser, microwave, or beta irradiation on CAs are under investigation (Cesario, Mahajan, and Shivkumar 2007). Biological cell‐based approaches, like the injection of autologous fibroblasts, have been proposed as an alternative method to create specific lesions and minimize distant injury (Bunch et al. 2006).

Pulse field ablation (PFA) is gaining attention as a novel energy source in cardiac ablation due to its tissue‐selective properties, primarily targeting cardiomyocytes while sparing surrounding tissues like nerves and blood vessels. However, one emerging concern is its potential to induce CA vasospasm, a transient but significant complication that can lead to ischemia. Unlike thermal ablation techniques, PFA uses non‐thermal energy, reducing the risk of thermal injury to CAs, but vasospasm remains a mechanism of CA injury that requires careful monitoring. Comparative studies suggest that the prevalence of coronary vasospasm with PFA is lower than the incidence of thermal damage seen with RF and cryoablation. However, further research is needed to fully understand the frequency and long‐term impact of this phenomenon, making it an important topic for future investigations in the field.

Finally, new approaches to biological or chemical ablation of cardiac tissue may offer more targeted elimination of cardiac myocytes while sparing major vascular structures.

Overall, a deeper understanding of CA pathophysiology after RFA is essential to enhance safety and further refine current ablation techniques.

## Author Contributions

Methodology, first draft and final draft: Syed Ali Raza Abidi; concept, first draft and final draft: Afreen Quadri; literature review, first draft, tables and images: Muhammad Umer Riaz Gondal; Tables, images, methodology and final draft: Fatima Hayat; first draft and final approval: Shafia Naeem; first draft and literature search: Fawad Talat; supervision and final approval: Amin Mehmoodi; concept, first draft and illustrations: Jahanzeb Malik.

## Conflicts of Interest

The authors declare no conflicts of interest.

## Data Availability

Data sharing is not applicable to this article as no new data were created or analyzed in this study.
